# Development and use of the PodEssential and Paeds-PodEssential triage tools to define “essential” podiatry services. A Delphi survey, scoping review, and face validity testing study

**DOI:** 10.1186/s13047-022-00525-8

**Published:** 2022-03-08

**Authors:** Cylie M. Williams, Alicia James, Sindhrani Dars, Helen Banwell

**Affiliations:** 1 Peninsula Health, 4 Hastings Road, Frankston, VIC 3199 Australia; 2 Monash University, School of Primary and Allied Health, 47-49 Moorooduc Hwy, Frankston, VIC 3199 Australia; 3 Allied Health and Human Performance, University of South Australia, North Tce, Adelaide, 5001 Australia

**Keywords:** Delphi technique, Podiatry, Risk, Triage, COVID-19

## Abstract

**Background:**

The coronavirus pandemic resulted in unique challenges for podiatrists in Australia. Podiatrists were tasked with having to make triage decisions about face-to-face care without clear guidelines. This research aimed to develop podiatry triage tools to understand individual risk for adults and children, and explore the face validity of both tools.

**Methods:**

An online three-round modified Delphi technique was used to elicit podiatrists’ opinions on conditions, assessments and social factors that elevate risk. Additional elements of known foot and/or leg risk were informed by a synchronous scoping review. Australian podiatrists who held a clinical role treating patients or directly managing podiatrists treating patients within the past six months were recruited.

Where 70% of participants reported the same or similar theme in Round 1, statements were accepted with consensus. Where 50–69% of participants reported a similar theme, these were returned to participants to rate agreement using a four-point Likert agreement scale. Statements identified in the scoping review were added at Round 2, if not already identified by participants. The final round presented participants with triage tools, and a series of mock patient scenarios.. Participants were asked to indicate if they would or would not provide face to face podiatry service based on these scenarios.

**Results:**

There were 40 participants who responded to Round 1 (Adult presentations), of these, 23 participants also provided paediatric presentation responses. Participants developed and agreed upon 20 statements about risk in podiatry service delivery for both adults and children across Rounds 1 and 2. The PodEssential and Paed-PodEssential were developed based on these statements indicating stand-alone condition risk (tier 1), elements that should elevate risk (in the absence of a stand-alone condition) (tier 2), and assessments results identifiying a limb at risk (tier 3) in adults and children respectively. Participants utilising these tools in Round 3 more frequently indicated face-to-face service when mock patient scenarios included a greater number elements, suggesting the tool can be useful in making triage decisions.

**Conclusion:**

The PodEssential and Paeds-PodEssential tools direct conditions requiring urgent attention as well as providing considered elements to a person’s health status to assist in making triage decisions.

**Supplementary Information:**

The online version contains supplementary material available at 10.1186/s13047-022-00525-8.

## Background

Podiatrists frequently need to make triage decisions about their patients. These decisions may be required to ensure patients meet overarching service provider or funding criteria, to manage wait lists or to comply with government directed emergency health orders. As such, it is important that triage outcomes are based on best available evidence to ensure those at greatest risk of adverse consequences are managed accordingly. However, in the absence of evidence-based tools and differences in service provision priorities, it is important that these decisions align with the nuances within clinical practice and be specific for the relevant health service setting.

Many medical and health professional groups have aimed to minimise face to face clinical service in response to the Severe Acute Respiratory Syndrome Coronavirus 2 (SARS-CoV-2) associated disease pandemic, or COVID-19 [1, 2]. In 2020 and 2021, public health and private health care settings world-wide were rapidly required to triage patients to minimise face-to-face service delivery [3, 4]. Similar to many countries, Australian podiatrists working across different settings often voluntarily reduced face-to-face services to minimise their own or staff risk [3], or were subject to government recommendations to provide ‘essential’ service only at specific times [4]. Many times, these public orders, have occurred without clear direction on what constitutes ‘essential’ service within the podiatry context and without the guidance of triage tools specific to pandemic based circumstances. Without clear guidance and professional consensus, this has resulted in challenging triage decisions for Australian podiatrists, managers of health services, and for peak bodies who play a role in supporting podiatrists to make triage decisions (e.g., Australian Podiatry Association).

This study aimed to develop a tool specific for podiatrists to guide decision making for what should constitute ‘essential’ services for adults and children seeking podiatry care, particularly in times of local, national or international crisis.

## Methods

### Design

The design involved a three-round modified Delphi survey method and a scoping review of the literature. Delphi methodology involves invited experts individually and anonymously being surveyed across sequential questionnaires (Rounds) for common consensus or agreement on a specific topic [5]. This method is considered an appropriate means of dealing with an absence of guidelines [6], allows flexibility in approach, in a modified manner, with the ability to be conducted online. To ensure robustness of this method, participants were asked to commit, and respond independently, for each round and review de-identified responses from the full participant group in subsequent rounds [7].

This Delphi survey consisted of three rounds; seeking consensus, agreement and face validity respectively. While this is consistent with similar podiatry-based Delphi surveys [8, 9], it was initially considered that agreement may require two rounds, with participants therefore consenting to a four-round survey. The initial round (Round 1) was a series of open-ended questions, where participants provided their individual opinions, to gather and assess the level of existing consensus amongst participants and inform further rounds. Round 2 sought participants agreement to the individual opinions delivered in Round 1 and presented findings from the literature not already identified by participants (as determined by the scoping review detailed below). Round two outcomes were then developed into two triage tools (PodEssential and Paeds-PodEssential respectively). These two tools were then tested for face validity in Round 3 [10].

Additional elements of known foot and/or leg risk were informed by a synchronous scoping review. Scoping reviews are an approach to for evidence synthesis and differ from a systematic reviews in purpose and aims. The purpose of a scoping review is to provide an overview of the available research evidence, without providing an answer to a suscient research question.

Scoping reviews are a useful tool in the overwhelming world of evidence. Scoping reviews also require rigorous and transparent methodology that ensures the reported information is accurate and trustworthy [11].

Monash University Human Research Ethics Committee (27514) approved this research.

### Participants

Australian podiatrists who held a clinical role treating patients or directly managing podiatrists treating patients within the past six months were eligible to participate. The survey was open to all eligible podiatrists to maximise participation and ensure a broad representation of skill mix and experience [7]. It was considered appropriate to consider eligible podiatrists as “experts” according to Delphi methodology due to their daily interaction, experience working in different settings, with different population and knowledge of clinical risk evaluation.

### Scoping review

A scoping review focused on peer reviewed articles and Australian health service guidelines detailing risk assessment, and prioritisation and/or triage of podiatry services was conducted concurrently and to add the robustness of evidence to Round 2. We used scoping review methodology to find relevant information [11]. Searches were conducted using Medline Ovid, CINAHL, EMBASE and AMED (Allied and Complementary Medicine) on the 12th of February 2021. We did not apply a date or study design limit to data searchers, but applied limits to human participants and English language limits were applied. The search strategy used within Ovid is available in Additional file 1: Appendix 1. A total of 16 articles met the inclusion criteria and were included for data extraction. Emails were also sent to podiatry departments of different health care settings around Australia requesting the triage tool/referral prioritisation information relevant to their departments. Inclusion criterion included reference to clinical decisions regarding who should be seen as part of clinical service provision. Sixteen peer review articles were identified from the search, and 28 triage tool documents were received from podiatry departments around Australia, that met the criteria were included for data extraction (Table 1).

**Table 1:** Extracted articles and grey literature tool characteristics to guide Round 2 questions

Study	Location	Description	Patient group	Clinical setting
Barshes [12]	N/A	Systematic Review	Patients at risk of Diabetic Foot Ulcers (DFUs)	Not applicable
Hughes [13]	UK	An online tool to determine when to seek help	People with symptoms of Raynaud’s disease	Online tool
Lavery [14]	USA	Case-control study	Patients with existing foot ulcer or history of foot ulcer	Not identified
Marmolejo [15]	USA	Review	Risks of developing Charcot neuroarthropathy foot	Not applicable
Antonopoulos [16]	Greece	Review	Patients with wounds after revascularization	Not applicable
Brechow [17]	Germany	Prospective study	Patients with existing diabetes related foot wounds	High Risk Foot Unity - multidisciplinary team in a hospital setting/outpatients and then transferred to outpatients
Elmarsafi [18]	USA	Retrospective study	Patients with diabetes who underwent osseous charcot reconstruction	Orthopaedic team/outpatients
Chamberlain [19]	USA	Literature review	Patients with a diagnosis of diabetes	Not applicable
Morbach [20]	Germany	Prospective study	Patient with a diagnosis of diabetes that develop ulcerations	Diabetic foot service
Jenkins [21]	UK	Scoping review	Predictors of poor wound healing	Not applicable
Ryan [22]	UK	Prospective audit	Impact of additional staff on patient’s with diabetes in community podiatry	Community podiatry - diabetic patient cohort
Bicer [23]	Turkey	Cross sectional study	Patients diagnosed with diabetes	Outpatient clinic at hospital
Alves [24]	Netherlands	Prediction model	Patients presenting to GP with joint pain	Primary Care
Krentz [25]	UK	Review	Patients with diabetes and peripheral vascular disease	Review
Yagihashi [26]	Japan	Cohort study	Patients with diabetes	Outpatients
Shih [27]	USA	Cohort study	Patient with diabetes	Hospital
Diabetes feet Australia and Australian Diabetes society [28]	Australia	Coronavirus: managing foot disease in the COVID crisis	People with Diabetes related foot disease	All
Queensland Health [29]	Australia	Minimum referral criteria	People with diabetes	All
Northern Health [30]	Australia	Podiatry triage guide	Acute inpatient podiatry referrals and Acute and sub-acute outpatient podiatry referrals	Public health inpatient and outpatient settings
National Diabetes Services Scheme [31]	Australia	Diabetes Foot Risk Stratification and triage	People at risk of Diabetes related foot complications	All
Victoria Paediatric Orthopaedic Network [32]	Australia	Paediatric orthopaedic referral guidelines	Children	Public hospitals with paediatric services
South West Healthcare [33]	Australia	Podiatry inpatient services (Warrnambool only)	Inpatients	Public hospital
South West Healthcare [34]	Australia	Podiatry new consumer and diabetic foot risk assessment and classification	General community	Public hospital and community health
Western District Health Service [35]	Australia	Primary and preventative health service guidelines	General community	Public hospital and community health
Caulfield community health service [36]	Australia	Podiatry for children	Children under the age of 18 in the general community	Community health
Caulfield community health service [37]	Australia	Podiatry	General community	Community health
Bass Coast Health [38]	Australia	Podiatry Priority Tool	General community	Community health
Cobram District Health [39]	Australia	Podiatry Services	General community eligibility defined by government funding guidelines	Community health
Moyne Health Services [40]	Australia	Podiatry Client Assessment, Classification and Care Planning Procedure	New patients referred into health service	Community health
Wimmera Health care [41]	Australia	Podiatry Department Service Prioritisation Clinical Guideline	Outpatients	Public hospital
Golburn Valley Health [42]	Australia	Podiatry referral guidelines for health professionals	General community eligibility defined by government funding guidelines	Community Health
Barwon Health [43]	Australia	Podiatry client ongoing categorization and care planning	General community	Community health
South Gippsland Hospital [44]	Australia	Podiatry services at South Gippsland hospital	General community eligibility defined by government funding guidelines	Community health
Peninsula Health [45]	Australia	Podiatry and Podiatry - Child	General community eligibility defined by government funding guidelines	Community health
Mallee Track Health and Community Service [46]	Australia	Podiatry procedure for aged care facilities	Residents of aged care facilities	Community Services
Cabrini [47]	Australia	Allied Health Referral, Triage and Management - Inpatients	Inpatients	Private hospital
Castlemaine health [48]	Australia	Podiatry services acute/subacute procedure	Inpatients	Public hospital
Swan Hill District Health	Australia	Podiatry Service Provision Protocol	General community	Community health
Lyndoch Living [49]	Australia	Podiatry Risk Assessment	Residents	Independent living facility
Terang and Mortlake Health Service [50]	Australia	Podiatry new client assessment, diabetes foot risk assessment and classification	General community	Community health
South West Healthcare [51]	Australia	Podiatry for children - information for teachers, parents and health professionals	General community	Community health
Monash Health [52]	Australia	Podiatry Acute and subacute inpatient priority tool	Inpatients	Public Hospital
Northern Sydney Local Health District [53]	Australia	Podiatry triage and waiting procedures	General community who meet prioritization triage matrix	Community health
Department of Health [54]	Australia	Community health priority tools	General community	Community health
Gippsland South Health Service [55]	Australia	Podiatry new patient risk assessment, stratification and treatment planning	General community	Community health

### Procedure

Podiatrists were recruited through institutional and personal social media accounts of the authors, in addition to national advertising through the Australian Podiatry Association and the Australian Foot and Ankle Research Network. Participants were provided with an information sheet and checked an online consent box for ongoing communication as part of the research. As part of this consent, it signified ongoing commitment to responses to all rounds. All survey rounds were conducted online and there was no intra-panel communication. Participants were requested to keep their responses confidential at each round.

Round 1 questions were specifically open-ended, aimed at determining consensus on which stand-alone conditions require urgent face-to-face care, existing or recent health or assessment outcomes that elevate risk for the individuals, and any other factors that may increase risk that require consideration when triaging patients. These questions were presented to participants through a purpose-built survey, developed by the authors. All authors hold podiatry-based clinical and academic positions and have extensive experience in survey development for this methodology [8, 56–58], which informed language used in questioning. Round 1 was piloted with two podiatrists external to the research team, and a non-health professional, with wording modified for clarity based on the feedback.

Round 2 statements were developed by outcomes of Round 1 and the synchronised scoping review. Specifically, risk elements that impact on triage decisions identified by the scoping review that had were not identified by participants in Round 1, were added to statements developed by participants for Round 2.

Round 3 was developed on the outcomes achieved from Round 1 and Round 2. Statements related to stand-alone conditions requiring urgent face to face care were incorporated into tier 1 and given an arbitrary score of 3 points per item. Statements related to assessment or health status factors that should elevate risk (in the absence of stand-alone conditions) were incorporated into tier 2 and awarded 2 points per item. Risk factors that may increase risk that require monitoring were placed in tier 3, at 1 point per item. These were then presented as two tools; the PodEssential tool to triage adults, and the Paeds-PodEssential aimed at triaging children attending podiatry services.

All data from each survey round were collected using the online survey platform Qualtrics® software (Qualtrics, Provo, UT, USA). The data were linked during the rounds through participant-provided email only. Rounds 1 and 2 were open for four calendar weeks and Round 3 was open for two weeks. Participants were reminded weekly to maximise retention of participants throughout the rounds.

All participants were offered their individualised responses and provided overall feedback at the end of each round. Results were provided to the participants completing all three rounds. Participants were incentivized through a competition process and optional entering a draw to win one of ten, $50 gift cards as recognition of the time spent completing each round.

#### Round 1

Participants were asked to provide their gender, state or territory of primary practice, information regarding their practice setting, recency of practice and if they treated children. This information was to ensure appropriate representation of the podiatry profession was attained through the sampling strategy and to guide the display of questions. Based on the information provided, questions were displayed using survey software logic. This meant that all participants received questions relating to adult presentations, however, questions pertaining to children’s presentations were only displayed to podiatrists who had provided clinical care for at least one child within the past six months.

Participants were asked to respond to a series of questions over three sections: specific clinical presentations; social factors that impact health and wellbeing; and what diagnostic tests or tools may additionally influence the risk of a patient requiring additional medical attention if unseen when referred or known to the practice. The full survey is in Additional file 2: Appendix 2.

Questions included:

1. *Thinking about podiatry related issues in adults, which presentations/conditions (as a stand-alone presentation) would lead you to triage someone as a high priority?*
2. *Now thinking about the factors that may increase an adult’s risk of complications. These factors may be the clinical signs/symptoms, suspected or actual conditions, lifestyle, psychosocial concerns* etc. *Please list factors that would lead you to consider someone at increased risk.*
3. *Thinking about podiatry related presentations/conditions in conjunction with risk, what (if any) further presentations/conditions or results of recent assessments (please be specific) in adults would lead you to elevate the triage for someone to be at a higher priority if you previously perceived them as being in a moderate or lower risk group?*

For participants who indicated they also saw paediatric patients, these three questions were repeated with the same phrasing, but the word ‘adult’ was replaced with ‘child or young person’.

Inductive quantitative content analysis of the open responses from Round 1 were undertaken to develop Round 2. This method of analysis allows the statements or comments to be considered individually, but then aligned against common themes [59]. This approach meant we considered the first participant’s comment and developed one or more statements from this. We then reviewed the comments from the next participants and either aligned them with the existing statements or generated new statement/s. We did this even when the response was a single word. This analysis took an iterative approach. If a new statement emerged, earlier comments were re-coded against this change. For example, where participants used words or phrases such as “LOPS (Loss of protective sensation)”, “neuropathy”, “painful neuropathy”, “pin and needles”, “foot pain making patient unable to work”, these were grouped under the one heading of “*Change in sensation at the foot and/or skin that results in pain, impacts on activities of daily living or protective sensation*”.

The data were initially analysed by a single researcher (AJ). To reduce individual bias, the comments and statements were then re-coded by an additional member of the research team (CMW). Disagreements were resolved by discussion and all statements presented to the authorship team for agreement. We acknowledge reflexivity being a concept that introduces personal bias into research [60]. For this team, this meant considering our experiences in triaging for podiatry in different public health services, university-based podiatry clinics and how this differs within the private practice setting.

Statements were accepted as reaching consensus where 70% of participants indicated the same themed statement, statements where 50–69% of participants indicated the same themed statement were reviewed by the participants in Round 2 to ensure adequate consideration, and statements where less than 50% of participants indicated the same concept were excluded from future rounds. Using this percentage was consistent with existing literature on the modified Delphi technique [8, 57].

#### Round 2

Statements requiring consideration for agreement were presented to participants in Round 2 along with additional statements directed from a synchronous scoping review that were not identified by participants in Round 2 also added (Additional file 3: Appendix 3).

Participants were then asked to consider each statement and indicate agreement on a four-point Likert scale (where 1 was Strongly Disagree, 2 was Disagree, 3 was Agree and 4 was Strongly Agree). Participants were also asked to provide suggestions to statement wording and comment further as desired. Comments received in Round 2 were managed in the same manner as comments received in Round 1.

#### Round 3

Round 3 was pre-planned to run as Round 4. However, as all statements had either been accepted or discarded in Round 2, face validity testing of the draft tools was conducted earlier than anticipated. Participants were initially provided with a restriction of service statement modelled on statements provided by Australian state and territory governments during limitations of allied health services as part of COVID-19 pandemic response “Podiatrists must use telehealth where possible and ONLY treat face-to-face if providing essential clinical care that cannot be delayed”. All participants were then provided with seven short referral cases with mock adult patient scenarios identifying a variety of conditions, complications, and risk factors. Participants previously responding to child focused questions in Round 1 and 2 also received seven short referral cases with mock paediatric patient scenarios. These mock patient scenarios were developed by the authors based on their public health and private practice experience. Participants were tasked with using the tool/s to triage each patient presentation as ‘essential’ (e.g., patient would meet the essential clinical care criteria enabling face-to-face service) or whether they would provide an alternative service via telehealth, defer face-to-face appointments until restrictions on services lifted, or remotely monitor for any status change via email or phone. Participants were also invited to provide comment on the use of the tool.

##### Analysis

In establishing the protocol, the authors made a priori decision of the Delphi concluding if the participant response rate dropped below 70%, or after face validity was tested. Participants who did not complete the entire questionnaire in Round 1 were excluded and not invited to complete subsequent rounds. Survey responses were exported, and all descriptive statistics and analysis of responses for each round was conducted, in Microsoft Excel 2018 (Microsoft Corp, Redmond Washington).

To examine face validity of the tool use, numerical scores were assigned by the author team after participants made a binary decision (Yes/No) to provide face-to-face clinical care on mock patient scenarios. This post-hoc decision to allocate numerical scoring was also made to understand if there was a hierarchical scoring pattern or cut-off point score based on types of conditions or patient presentations to inform future research into the development of a scoring system. The author team allocated scoring system of the mock participants was graphically displayed together with the frequency (%) of participants indicating they would provide face-to-face care of the mock patient based on their use of the tool to guide their decision to understand if there was a scoring cut point based on the mock patient’s estimated level of risk.

## Results

**Fig. 1:**
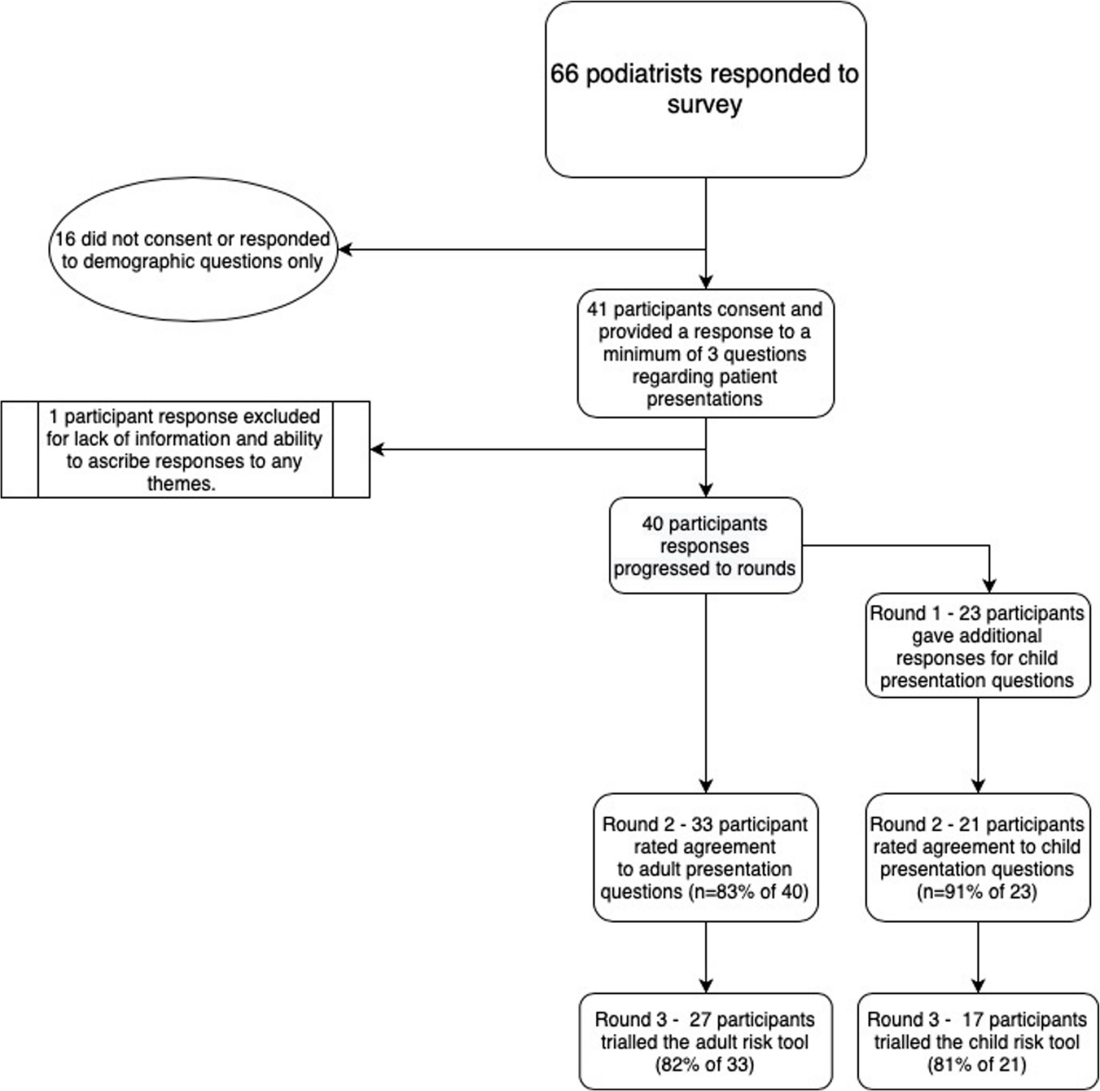
Flow of participants through the three rounds

There were 66 podiatrists who responded to advertising about this researh and entered the survey. After removal of non-consent and demographic only response information, 40 participant responses were included in Round 1 for adult presentations. Of these 40, 23 also provided additional paediatric presentation responses. Table 2 provides summary demographics of participants in Round 1 and Fig. 1 provide details of the retention of participants through the three rounds. There was an 83% (33 of 40 participants) retention rate for Round 2, and 68% (27 of 40 participants) response rate to trialling the tool in Round 3.

**Table 2:** Summary demographics of participants (*n* = 40)

	n (%)
Gender (Female)	34 (85%)
**State or territory**	
*Victoria*	9 (23%)
*New South Wales*	8 (23%)
*Queensland/Tasmania/Western Australia*	7 (18%)
*South Australia*	10 (25)
Practice setting	
*100% Private practice with or without management time*	23 (58%)
*100% Hospital or community health/blended public role*	6 (15%)
*Blended public health, private or education role*	11 (27%)
Recency of practice	
*< 5 years of practice*	5 (12%)
*5–10 years*	8 (20%)
*11–15 years*	4 (10%)
*> 15 years*	23 (58%)

There were 54 statements provided by participants in Round 1, eight met consensus and nine were returned to particulars for agreement in Round 2. There were five statements identified in addition to the participant statements through the synchronous scoping review for participant rating of agreement in Round 2 (Table 3 and Table 4).

**Table 3:**
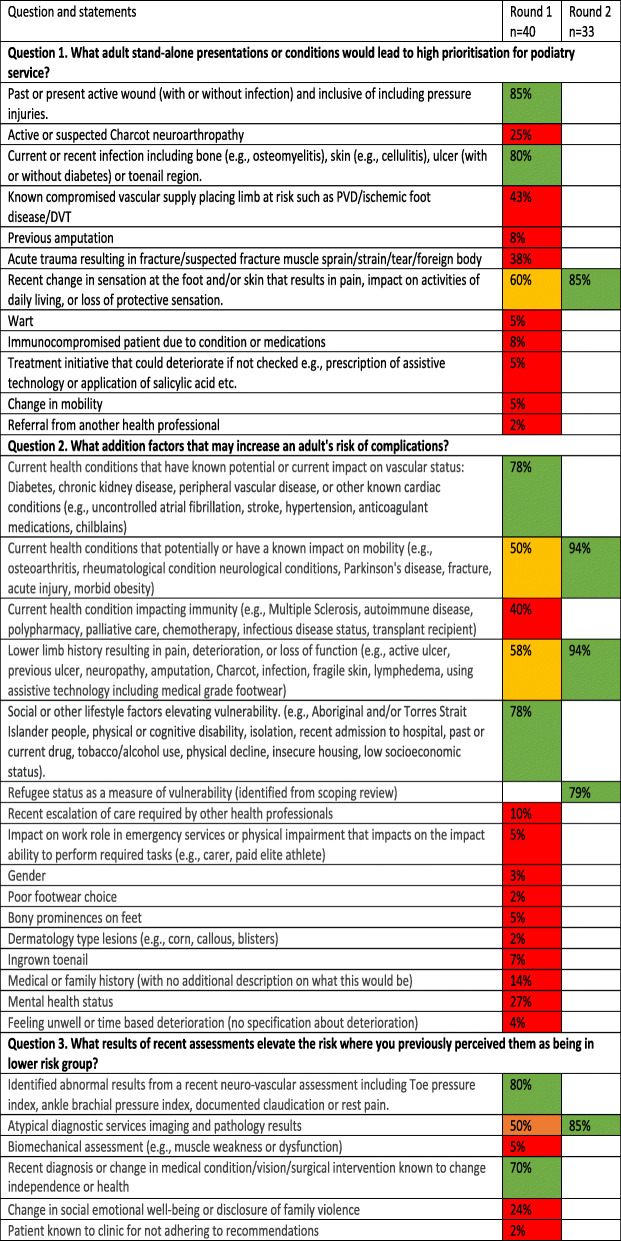
Statements generated based on adult presentations where red shading indicates the statement did not progress, orange shading indicates progression to the next round or green shading indicates consensus or agreement during that round

**Table 4:**
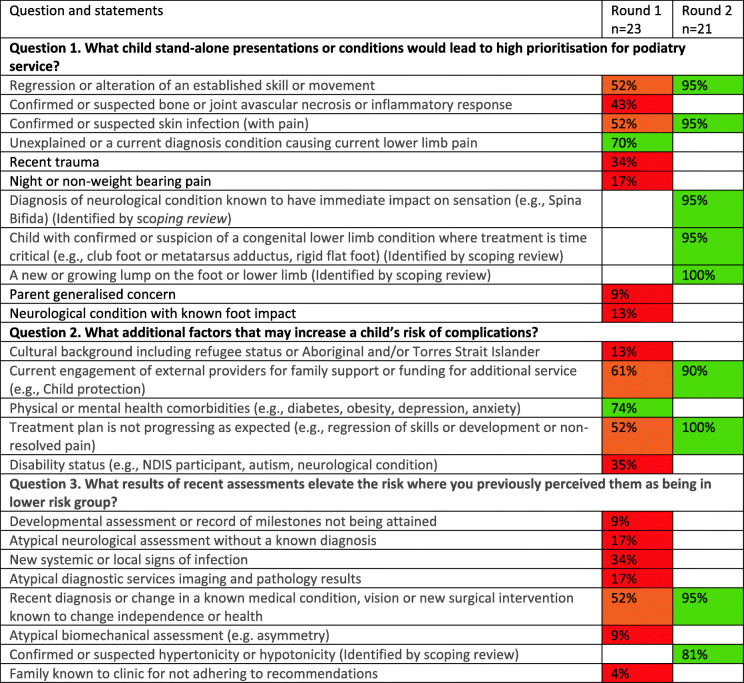
Statements generated based on paediatric presentations where red shading indicates the statement did not progress, orange shading indicates progression to the next round or green shading indicates consensus or agreement during that round

**Fig. 2:**
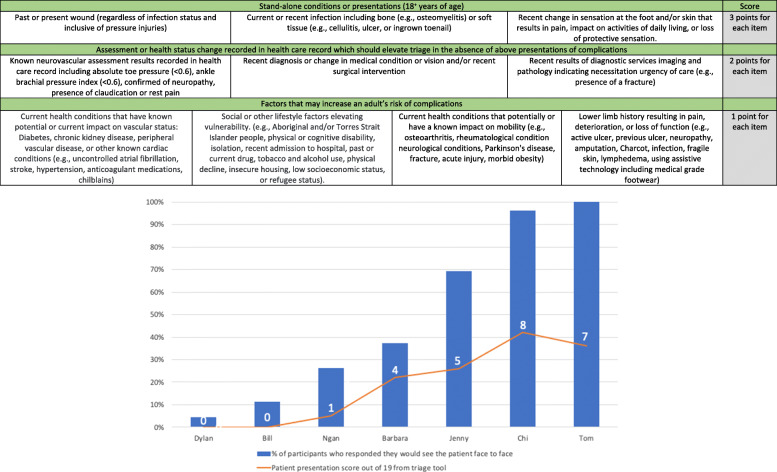
PodEssential Adult triage tool and graphical presentation of the number of participants who indicate they would see the patient face-to-face and the scores for the seven patient presentations

**Fig. 3:**
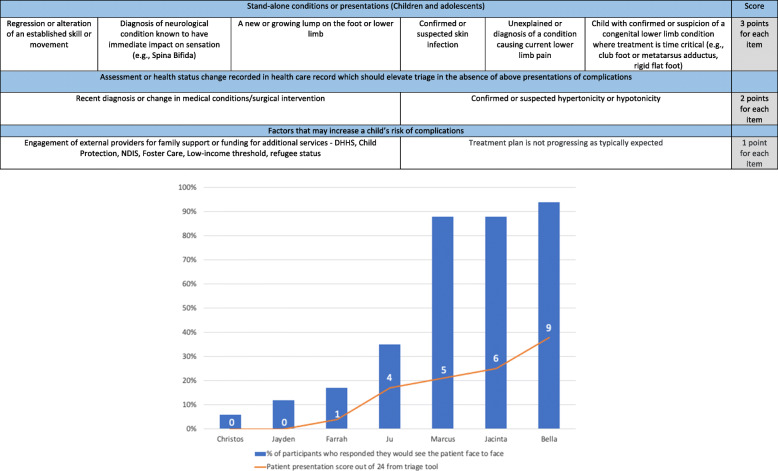
Paed-PodEssential Child triage tool and graphical presentation of the number of participants who indicate they would see the patient face-to-face and the scores for the seven patient presentations

These statements were refined into the PodEssential triage tool to support decisions for adult and the Paed-PodEssential for paediatric presentations with the post-hoc scoring applied to individual items identified by participants. Participants used the developed tool in Round 3. A summary of how participants responded to the mock patient summaries and a graphical relationship to face-to-face service are supplied in Figs. 2 and 3. The increase in score aligned with higher proportion of participants indicating they would see the patient face to face, but no apparent cut-point score emerged on where all participants agreed. Additional file 4: Appendix 4 provides in depth detail of the mock patient scenarios, elements of essential service, and participant response numbers. Participants comments were primarily in support of the tool. Some highlighted examples where a patient may have met the criteria for face-to-face services based on pain impacting activities of daily living, however, they felt they could still provide ample podiatry service via telehealth. Others described a preference to defer as much face-to-face care as possible despite the patient presentation being categorised as essential through the tool statements. These comments were only made by participants who practiced in Victoria. Conversely, one participant practicing in Western Australia adopted a “see all” approach regardless of if they had an identified element of essential need from tool or not.

## Discussion

This research developed a triage or decision support tool for adult and child podiatry patients using the experience of podiatrists’ and their experience within different settings, and informed by evidence. The use of this methodology was considered appropriate due to breadth of clinical guidelines determined in the scoping review, and the deficit of published triage tools that than those for identification of critical limb loss or risk of death relating to limb disease. Delphi survey methodology is a valid and reliable way to gather expert opinion when there is scarce evidence on a clinically important topic [6]. This is the first tool development taking into account not just risk relating to critical limb loss, but a broad approach to other conditions or presentations that place a person at risk of having to seek care from an alternative health care provider. This element is unique to this research.

The use of PodEssential and Paed-PodEssential could be adapted within a variety of practice settings, particularly where triage is required based on funding models, such as those common to Australia in both public and private practice. These funding models often take into consideration personal care needs to minimise escalation to public health services, disability support or packages that aim to keep older adults independent at home. These tools could aid podiatrists in decisions to ensure those at greater need can access to podiatry care. Importantly, the tool may be considered adaptive, and person-centred, not just high-risk foot specific. Participating podiatrists considered the impact foot pain has on people’s ability to carry out their activities of daily living and factors that make a person more vulnerable to poor health outcomes and escalating health care needs as a result. This can offer meaningful direction for podiatrists required to triage patients outside of pandemic-based circumstances as well.

High risk foot presentations are by far the most emergent presentation to podiatrists [28]. Those at highest risk of limb loss are usually managed within the Australian hospital or public health care setting [29]. When a wound has healed, a person may then have their care transferred to private podiatry settings [29, 45]. While some high risk conditions were not named in this tool based on consensus, many of these individual risks or diagnosis aligned with the overarching categories developed through the themeing process, rather than individually called out. For example Charcot neuroarthropathy, while not meeting consensus as a stand alone condition, would still be considered in the grouping of loss of protective sensation or potentially the grouping where someone who had a history of a foot wound. Another example where a condition did not meet consensus, was for those who have known peripheral vascular disease. While not identified as a stand alone condition within the tool, patients with this condition would considered captured within the tool element of having conditions known to impact vascular status and those who have assessment with known vascular deficiency. Given the breadth of individual conditions, a check box list could have been an alternative, however as per the Delphi methodology, many would not have made consensus. In utilising overarching categories, those individual conditions at higher risk of limb loss identified within the scoping review were still captured as essential and treated in both public and private practice.

Both of these practice setting in Australia were impacted to varing degrees during the COVID-19 lock-down orders that were provided with the most varied advice on what patients were and were not permissible to be seen face-to-face in Australia [4]. Similarly, these were also the locations with the large COVID-19 community numbers and podiatrists reporting their own preferences for face-to-face service [3]. Podiatrists lived experience in working with Australian government’s Department of Health advice on restricting services may have influenced the face validity testing. For example, at the time of data collection, podiatrists in Western Australia and Queensland had consistently very low community COVID-19 transmissions with very limited service restrictions, therefore, may not have deeply considered the public health implications of face-to-face orders in other states. This meant the face validity task was more hypothetical for them. Whereas podiatrists in Victoria had experienced long-term restrictions, with high community cases and three waves of outbreaks during 2020–2021, were more supportive of triage away from face-to-face services and responded to the face validity testing accordingly [61]. Podiatrists in Victoria, and to some extent New South Wales, are also more versed in checking and interpreting terminology used by government to limit public movements to a greater extent, than those in other jurisdictions [4, 62].

Unfortunately, terminology utilised by the various Australian states and territories health departments and government bodies has not been consistent throughout 2020–2021, and this continued during data collection. This has potentially created understandable differences in interpretations of “essential”, “urgent” or “critical” care as no specific definitions were provided for the different health and allied health providers. This may have further challenged podiatrists responding to this study, as some podiatrists only provided responses relating to “high-risk” foot presentations without considering other factors that elevate a patient’s risk of complications or ability to perform activities of daily living. The later being appreciated by some podiatrists and their inclusion of essential care also including factors such as someone’s heel pain and it’s impact on their ability to work. Despite these challenges, most podiatrists strongly supported alternate care options over face-to-face service. Many of our participants opted for alternative care even when the triage tool suggested the presenting complaint was potentially permissible under the “essential care only” direction. The use of telehealth and remote monitoring of different presentations during 2020–2021 has been variable for many podiatrists across the country, some gaining extensive experience, while others have not engaged at all [2, 63]. This is similar to what has been seen within other allied health professions [2, 63]. Again, this may be related to need and experience. For example, podiatrists in Melbourne (Victoria) had over six months of minimal face to face services within the last two-year period, compared to those working in other states and territories such as Western Australia, South Australia, Northern Territory and Tasmania who had very limited restrictions to practice by comparison [4]. It was apparent, this lived experience had a strong influence on the results and interpretation of triage. The challenge for podiatrists is to be consciously aware of our individual experiences when it comes to determining ‘what is essential’ in podiatry practice. The tools developed in this study remind us that health and foot pathologies can change over time, and the variable durations of the COVID-19 restrictions to practice orders can also impact our thoughts on offering face-to-face service or alternatives. These triage tools should not be used in place of good clinical judgement or where there is evidence to support additional clinical care, instead it should support clinicians in justifying their models of care during limitations on face-to-face service or determining who should be seen earlier if there are limited services.

This research was limited by its methodology and being based on expert opinion. It is also to acknowledge that while tools are important to support clinical decision making, all presentations (e.g., diagnosis, social setting, complexity) may not have been captured. The use of the Delphi technique in the context of evidence-based practice constitutes low level evidence. Additionally bias of the researchers may have been introduced during theming the statements. The research team had various triage experience (< 1 year through to > 25 years) and held each other to account through meetings about theme development. We attempted to minimise bias through transparency of themes to participants. We also provided opportunities at each round for participants to refute or question where there was ambiguity. Additionally, remaining anonymous and keeping statements confidential are suggested requirements of participants in the Delphi technique [6]. This requirement is to minimise the effects, if any, of collusion on the results. It cannot be guaranteed that participants remained anonymous to their peers given the small size of the podiatry profession. All participants were cautioned to keep both their responses and participation confidential to minimise this bias risk. Lastly, we also acknowledge that in naming the mock patients in research team developed scenarios, we may have introduced unconscious bias towards gender or cultures that may have factored into treatment decisions. We anticipate all podiatrists were able to focus on the intent of the research, this is a known limitation in decisions making [64].

There are numerous benefits for using a single triage tool and developing it with this methodology. The utilisation of a concise evidence-based tool designed by the podiatry profession allows for government, policy makers, health services and associations to be confident in the healthcare messaging and services being offered across the country. In the absence of overarching guidelines, development of such tools through Delphi methology allows for rapid consensus, which is commonly needed in times of crisis or when rapid decisions need to be made. The implementation of these triage tools into clinical practice will allow for practitioners to be confident in their models of care, service provision and advertisement to the public if there are future restrictions on practice as a result of public health directives. These tools may also assist where triage decisions are required based on staff shortages or long waiting lists. These tools may support podiatrists to consider the information they should collect from the patient, or review in their clinical notes, to ensure they see those most at risk when needing to make triage decisions. Furthermore, when triage is often completed in public health ambulatory services or busy private practices it is often by skilled administration staff. This type of tool may provide opportunities to reduce burden on health professionals, allowing more time for care provision rather than administrative burden. Future research should consider using the tool with the scoring template to understand cut points that place a patient at greater risk of escalation of care needs and/or the reliability in use of the tool with or without a health professional completing it. This triage tool may also have international implications for podiatrists who work in similar practice settings such as the United Kingdom or Canada. This triage tool may also have international implications for podiatrists who work in similar practice settings such as the Unitied Kingdom or Canada. Researchers should consider localised small group validation when considering the use of these tools internationally to understand cross-cultural validity and the appropriateness of terminology.

## Conclusion

This podiatry triage tool could be used to support decisions to provide face-to-face service where there are directives allowing podiatrists to provide essential care only. Podiatrists using this tool should also reuse the tool should patient’s circumstances change or there are extended timeframes where face-to-face services should be delayed.

## Supplementary Information

**Additional file 1.** **Appendix 1:** Search strategy and PRISMA flowchart.

**Additional file 2. Appendix 2: **Round 1 questions.

**Additional file 3. Appendix 3: **Round 2 questions.

**Additional file 4.** **Appendix 4:** Patient scenarios presented with highlight elements aligning with triage tool.

## Data Availability

All available data is provided within the manuscript.

## Abbreviations

SARS-CoV-2: Severe Acute Respiratory Syndrome Coronavirus 2
n: number
