# Supplementary material for: Development and use of the PodEssential and Paeds-PodEssential triage tools to define “essential” podiatry services. A Delphi survey, scoping review, and face validity testing study
Source: J Foot Ankle Res. 2022 Mar 8;15:20. doi: 10.1186/s13047-022-00525-8 (PMC8901864; doi:10.1186/s13047-022-00525-8)
Supplement: Supplementary file 1 — Additional file 1. Appendix 1: Search strategy and PRISMA flowchart. [file 13047_2022_525_MOESM1_ESM.pdf]

Appendix 1: Search strategy and PRISMA flowchart

1. client*.mp. OR
2. service user*.mp.
3. patient*.mp.
4. Patient Selection.mp.
5. 1 or 2 or 3 or 4
6. categor*.mp.
7. setting.mp.
8. strateg*.mp.
9. system*.mp.
10. tool*.mp.
11. Classification.mp
12. 6 or 7 or 8 or 9 or 10 or 11
13. queu*.mp.
14. delay*.mp.
15. waiting time*.mp.
16. "wait time".mp.
17. waiting list*.mp.
18. wait list*.mp.
19. waitlist*.mp.
20. Systems Theory.mp.
21. 13 or 14 or 15 or 16 or 17 or 18 or 19 or 20
22. priorit*.mp.
23. triag*.mp.
24. Health Priorities.mp.
25. Triage.mp.
26. risk.mp
27. podiatr*.mp.
28. chiropod*.mp.
29. foot.mp.
30. feet.mp.
31. leg.mp.
32. lower limb.mp.
33. ankle.mp
34. 27 or 28 or 29 or 30 or 31 or 32 or 33
35. 22 or 23 or 24 or 25 or 26
36. 5 and 12 and 21 and 34 and 35
37. limit 36 to yr="2000 -Current”

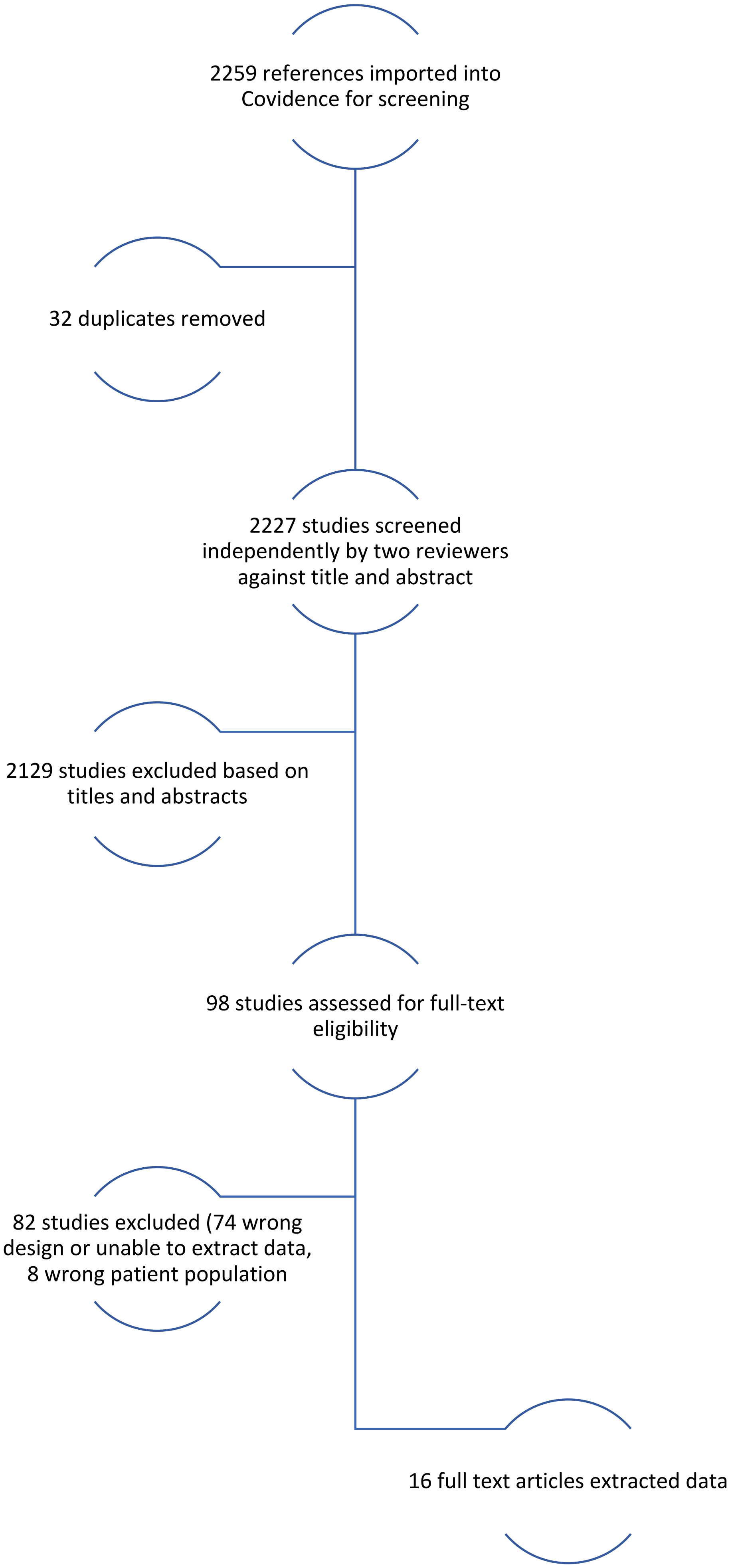
2259 references imported into Covidence for screening
32 duplicates removed
2227 studies screened independently by two reviewers against title and abstract
2129 studies excluded based on titles and abstracts
98 studies assessed for full-text eligibility
82 studies excluded (74 wrong design or unable to extract data, 8 wrong patient population
16 full text articles extracted data
