# Supplementary material for: Development and use of the PodEssential and Paeds-PodEssential triage tools to define “essential” podiatry services. A Delphi survey, scoping review, and face validity testing study
Source: J Foot Ankle Res. 2022 Mar 8;15:20. doi: 10.1186/s13047-022-00525-8 (PMC8901864; doi:10.1186/s13047-022-00525-8)
Supplement: Supplementary file 2 — Additional file 2. Appendix 2: Round 1 questions. [file 13047_2022_525_MOESM2_ESM.pdf]

INTRO/CONSENT

Project title: Developing a risk matrix for triage into podiatry services - a modified Delphi study
Project ID: 27514

Research team:
Dr Alicia James: Alicia.james1@monash.edu
Dr Helen Banwell: Helen.Banwell@unisa.edu.au
Ms Sindhrani Dars: darsy009@mymail.unisa.edu.au
A/Prof Cylie Williams: cylie.williams@monash.edu

Why are we doing this research?

- To find out how podiatrists make clinical decisions about the risk status of people who seek care from podiatrists.
- To develop clinical risk allocation guidance

Who can participate?

You can participate if you are:

- an Australian Podiatrist
- have treated patients or managed podiatrists who treated patients within the past 6 months.

This research is funded by the Australian Podiatry Research Education Foundation.

What do I have to do?

If you would like to participate:

- We will ask you for consent to participate and provide your email.
- Complete three-four (3-4) rounds of surveys, that will get quicker each round. The first survey will take up to 30 minutes, and subsequent surveys are expected to take up to 15 minutes.
- Survey rounds will occur over 3-4 months.
- You have the option to provide your details to go into a draw to win one of 10, $50 Coles Myer vouchers.

What happens if I don't want to finish the survey?

If you wish to stop the survey:

- You can close the browser window at any time.
- Any data that you have provided will be included in the research.

What will happen to my answers?

- Your answers will be stored in line with Australian Privacy Regulations.
- After everyone has finished the survey rounds we will write a report that will be published. Your answers in this report will stay private.

Risks and Benefits to participation

- We anticipate the only risk is that of inconvenience due to time. We hope the findings will assist clinical risk allocation in the future

---

Should you have any concerns or complaints about the conduct of the project, you are welcome to contact the Principal Investigators or the Executive Officer, Monash University Human Research Ethics Committee (MUHREC), depending on your concern:

Questions

Principal Investigators:
Dr Alicia James & A/Prof Cylie Williams
School of Primary and Allied Health Care, Monash University
email: alicia.james1@monash.edu Ph: 03 9784 8100
email: cylie.williams@monash.edu Ph: 03 9904 4344

Complaints:

Executive Officer
Monash University Human Research Ethics Committee (MUHREC)
Room 111, Chancellery Building E,
24 Sports Walk, Clayton Campus
Research Office
Monash University VIC 3800
Tel: +61 3 9905 2052
Email: muhrec@monash.edu
Fax: +61 3 9905 3831

This research has been approved by the Monash University Human Research Ethics Committee.

For more detailed information about this research please click on the below link.
Long explanatory statement

To consent, and participate in this research, please enter your email.

By entering, you are confirming you are a podiatrist that meets the inclusion criteria and confirm that you have read the above information about the study. You also understand that participation involves in taking part in the **three-four online Delphi panel** rounds to the best of your ability. These will be 4-6 weeks apart.
You also agree to keep your involvement and information developed through this process confidential.

Please provide your email below, this will be the email we contact you for repeat surveys, link your answers and provide you with a final report.
Please provide an email that you regularly access. This will be the email we use to link your data:

ABOUT YOU

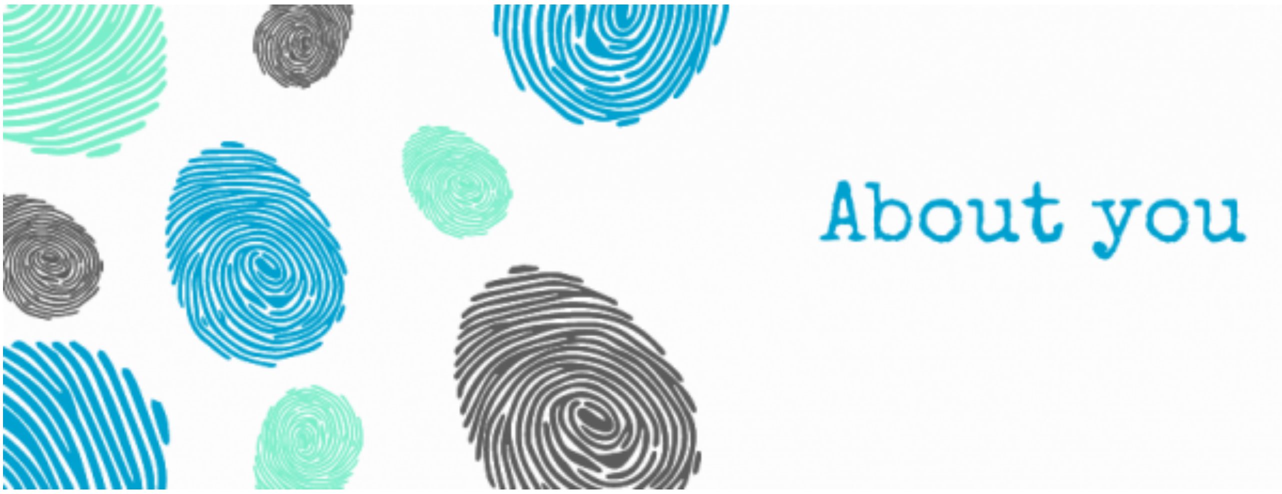

Your gender?

- ○ Female
- ○ Male
- ○ X (Indeterminate/Intersex/Unspecified)

Primary practice state/territory

- ○ Victoria
- ○ South Australia
- ○ Tasmania
- ○ Australian Capital Territory
- ○ Queensland
- ○ Western Australia
- ○ New South Wales

Percentage of time spend in the following practice settings over most recent usual working month (enter in a number % to add up to 100)

Private practice [0]

Community Health

Hospital (acute or subacute)	0
Supervision of students in a student clinic	0
Management/academic (patient related)	0
Management/academic (non-patient related)	0
Total	0

Years since first registered as a podiatrist

- <5 years
- 5-10 years
- 11-15 years
- >15 years

Have you assessed or treated at least one (1) patient aged under 18 in the majority of usual working weeks for the past 6 months?

- Yes
- No

As health professionals we often have to triage patients. To do this, we generally consider the presentation/condition that the person is seeking help for, and any factors that increase their risk of complications. We want to unpack this below to work out what 'stand-alone' podiatry related presentations (condition) result in a patient being categorised as high risk or a higher priority, and what other factors (e.g. risk) may lead us to triage someone as needing to be seen earlier (over someone with the same condition) and what test results or changes to health status may change the level of risk.

When you are answering the following questions, please think about the clients/patients

you have seen over the past 6 months and answer in terms of what your '*best practice*' options would be (e.g., do not consider availability, travel requirements or pandemic-related restrictions in your answer).

Please note we have a separate section for adults (the definition for this research is for people who are 18 years of age or older) or for children and young people (under the age of 18 years). If you responded as not seeing many children or young people, the paediatric questions won't be displayed.

Thinking about podiatry related issues in **adults**, which presentations/conditions (as a stand-alone presentation) would lead you to triage someone as a high priority?

Now thinking about the factors that may increase an **adult's** risk of complications. These factors may be the clinical signs/symptoms, a suspected or actual conditions, lifestyle, psychosocial concerns etc.
Please list factors that would lead you to consider someone at increased risk.

Thinking about podiatry related presentations/conditions in conjunction with risk, what (if any) further presentations/conditions or results of recent assessments (please be

specific) in **adults** would lead you to elevate the triage for someone to be at a higher priority if you previously perceived them as being in a moderate or lower risk group?

Now thinking about **children and young people**, please list any specific stand-alone presentations/conditions (not already listed previously) above that might lead you to triage them as a high priority.

Now thinking about the factors that may increase a **child or young person's** risk of complications**.** These factors may be the clinical signs/symptoms, a suspected or actual conditions, lifestyle, psychosocial concerns etc.
Please list factors that would lead you to consider someone at increased risk.

Thinking about podiatry related presentations/conditions in conjunction with risk, what (if any) further presentations/conditions or results of recent assessments (please be

specific) in **children or young people** would lead you to elevate the triage for someone to be at a higher priority if you previously perceived them as being in a moderate or lower risk group?

End

Thank you for your views. We will contact you via email in 4-6 weeks from the close of this survey. The next survey will ask you rate your agreement to various statements that have arisen from your and other people's responses in addition to the results from our literature review. The next survey will be much quicker for you to answer.

It is important to meet the aims of this research that you keep taking part in each round, so we thank you for your continued involvement.

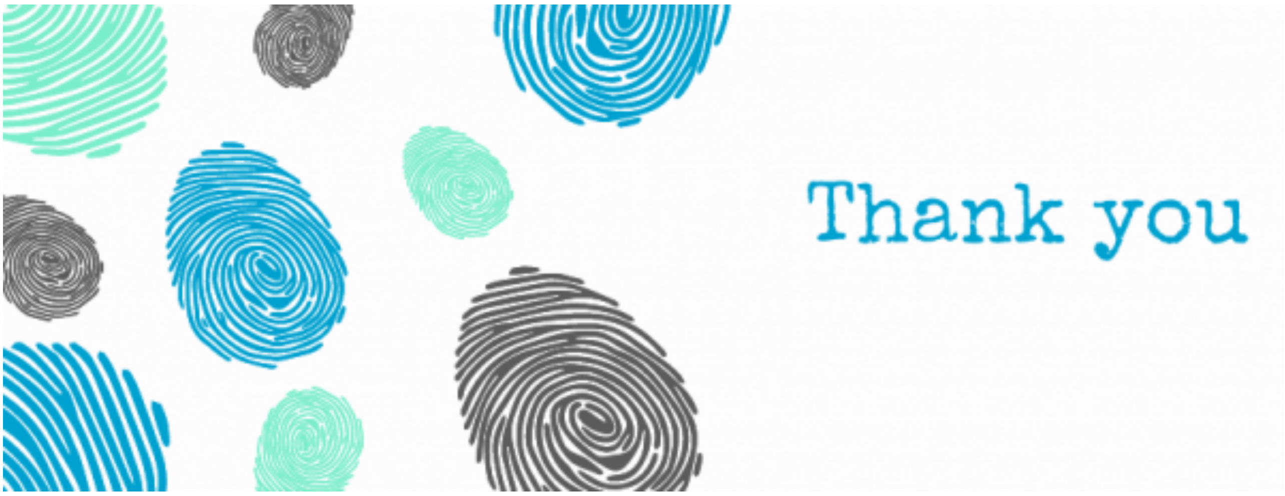

Powered by Qualtrics
