# Supplementary material for: Development and use of the PodEssential and Paeds-PodEssential triage tools to define “essential” podiatry services. A Delphi survey, scoping review, and face validity testing study
Source: J Foot Ankle Res. 2022 Mar 8;15:20. doi: 10.1186/s13047-022-00525-8 (PMC8901864; doi:10.1186/s13047-022-00525-8)
Supplement: Supplementary file 3 — Additional file 3. Appendix 3: Round 2 questions. [file 13047_2022_525_MOESM3_ESM.pdf]

INTRO/CONSENT

Monash University

University of South Australia

Peninsula Health

Round 2

What does my participation involve in this round?

We have collated the responses from the first round and now ask you to consider the outcomes and rank your agreement.

Firstly, here's what the last round gave us:
We had 41 podiatrists take part in helping define and describe what clinical presentations and patient circumstances are considered when triaging patients for care. There were 23 podiatrists also responding to the paediatric patient triage questions.

Where 70% or greater of all podiatrists said the same thing, a consensus was achieved and we included the name, condition or description, and will present this to you in this survey for your information.
Where 50%-69% of podiatrists responded the same, we have included it as a

statement for you to rate how much you agree. Where less than 50% of podiatrists responded similarly, this has not been included in this round.

Other information that is informing this round
We also completed a scoping review of the literature and podiatry triage tools in Australia to determine any other factors that have been identified elsewhere that impact on risk. These have been included for you to rate your agreement as appropriate.

To meet the aim of this research, it is very important you complete this survey to give us your opinion of the statements. It should take under 10 minutes.

If you would like a copy of your original responses in Round 1, please contact Cylie: cylie.williams@monash.edu

Please provide your email below so we can track responses and link them between groups in each round. Please use your same email for each round.

Adult

You were asked to consider the adult population for triaging. Consensus was reached on the following stand alone conditions or presentations for high priority triage:

1. Past or present wound (regardless of infection status and inclusive of pressure injuries)
2. Current or recent infection including bone (e.g., osteomyelitis) or soft tissue (e.g., cellulitis, ulcer or ingrown toe nail)

When you were asked to consider the adult population for high priority triaging, the following statement was consistent from the majority of podiatrists (50% - 69%).

Please utilise the following scale to rate your agreement of the following conditions/presentations (as a stand alone presentations) would meet the threshold for high priority triage.

	Strongly disagree	Disagree	Agree	Strongly Agree
Change in sensation at the foot and/or skin that results in pain, impact on activities of daily living or loss of protective sensation.	○	○	○	○

If you disagree with the above statement, please provide your reason or alternative wording suggestions.

You were asked to list the factors that may increase an adults risk of complications (including clinical signs, symptoms, a suspected or actual condition, lifestyle or psychosocial concerns). Consensus was reached on the following:

1. Current health conditions that have known potential or current impact on vascular status: Diabetes, chronic kidney disease, peripheral vascular

disease or other known cardiac conditions (e.g., uncontrolled atrial fibrillation, stroke, hypertension, anticoagulant medications, chilblains)

2. Social or other lifestyle factors elevating vulnerability. (e.g., Aboriginal and/or Torres Strait Islander people, physical or cognitive disability, isolation, recent admission to hospital, past or current drug, tobacco and alcohol use, physical decline, insecure housing, low socioeconomic status,).

The following factors were described by 50-69% of podiatrists or identified from the scoping review.

Please utilise the following scale to rate your agreement of the following conditions/presentations would increase an adults risk of complications

	Strongly disagree	Disagree	Agree	Strongly Agree
Current health conditions that potentially or have a known impact on mobility (e.g., osteoarthritis, rheumatological condition neurological conditions, Parkinson's disease, fracture, acute injury, morbid obesity)	○	○	○	○

Lower limb history resulting in pain, deterioration or loss of function (e.g., active ulcer, previous ulcer, neuropathy, amputation, Charcot, infection, fragile skin, lymphedema, using assistive technology including medical grade footwear)	○	○	○	○
Refugee status indicative of vulnerability (Scoping review)	○	○	○	○

If you disagree with any of the above statements, please provide your reason or alternative wording suggestions.

When asked to think about the adult population when assessing risk what podiatry assessments or results were likely to elevate the triage for someone to be at a higher priority (e.g., if you previously perceived them as being at a moderate risk, you may now consider them high risk)?

The following assessments reached consensus amongst the responding clinicians:
1. Neurovascular assessments including: Absolute toe pressure, ankle brachial pressure index, confirmation of neuropathy, presence of claudication, rest pain, loss of protection sensation
2. Wound Assessment or development of wound
3. Recent diagnosis or change in medical condition/vision and/or recent

surgical intervention

The following assessment results were rated as being used to elevate risk status by 50%-69% of podiatrists.

Please utilise the following scale to rate your agreement of the following assessments/results that would increase priority when being triaged:

	Strongly Disagree	Disagree	Agree	Strongly Agree
Results of diagnostic services imaging and pathology	○	○	○	○

If you disagree with the above statement, please provide your reason or alternative wording suggestions.

Are there any further comments you would like to make regarding adult presentations, conditions or assessment outcomes that would elevate someone to require high priority triage?

Paediatrics

You were asked to consider paediatric conditions or stand alone presentations for triaging. There were no statements meeting consensus for stand alone conditions or presentations for high priority triage.

The following stand alone conditions or presentations were described by 50-69% of podiatrists or identified from our scoping review.

Please utilise the following scale to rate your agreement with the following stand alone conditions or presentations that would meet the triaging threshold for high priority

	Strongly disagree	Disagree	Agree	Strongly Agree
Regression or alteration of an established skill or movement	○	○	○	○
Confirmed or suspected skin infection	○	○	○	○
Unexplained or diagnosis of a condition causing current lower limb pain	○	○	○	○
Child with confirmed or suspicion of a congenital lower limb condition where treatment is time critical (e.g., club foot or metatarsus adductus, rigid flat foot) (Scoping review)	○	○	○	○

	Strongly disagree	Disagree	Agree	Strongly Agree
Diagnosis of neurological condition known to have immediate impact on sensation (e.g., Spina Bifida) (Scoping review)	○	○	○	○
A new or growing lump on the foot or lower limb (Scoping review)	○	○	○	○

If you disagree with any of the above statements, please provide your reason or alternative wording suggestions.

You were asked to list the factors that may increase a child's risk of complications (including clinical signs, symptoms, a suspected or actual condition, lifestyle or psychosocial concerns). Consensus was reached on the following statement:

1. Physical or mental health conditions placing the child at risk of further complications (e.g., diabetes, morbid obesity, mental health conditions)

The following factors were rated by 50%-69% of podiatrists.

Please utilise the following scale to rate your agreement with the following factors increasing a child's risk of further complications

	Strongly disagree	Disagree	Agree	Strongly Agree

Engagement of external providers for family support or funding for additional services - DHHS, Child Protection, NDIS, Foster Care, Low-income threshold, refugee status	○	○	○	○
Treatment plan is not progressing as typically expected	○	○	○	○

If you disagree with any of the above statements, please provide your reason or alternative wording suggestions.

You were asked to think about the paediatric population when assessing risk what podiatry assessments or results were likely to elevate the triage to be at a higher priority (e.g., where you previously perceived them being in a lower risk group).

There were no statements that met consensus.

The following assessments or results were described by 50-69% of podiatrists or identified from the scoping review.

Please utilize the following scale to rate your agreement increase in priority when being triaged:

Strongly Strongly

	disagree	Disagree	Agree	Agree
Recent diagnosis or change in medical conditions/surgical intervention	○	○	○	○
Confirmed or suspected hypertonicity or hypotonicity (scoping review)	○	○	○	○

If you disagree with any of the above statements, please provide your reason or alternative wording suggestions.

Are there any further comments you would like to make regarding paediatric presentations, conditions or assessment outcomes that would, in your opinion, elevate someone to require high priority triage?

Powered by Qualtrics
