# Supplementary material for: Development and use of the PodEssential and Paeds-PodEssential triage tools to define “essential” podiatry services. A Delphi survey, scoping review, and face validity testing study
Source: J Foot Ankle Res. 2022 Mar 8;15:20. doi: 10.1186/s13047-022-00525-8 (PMC8901864; doi:10.1186/s13047-022-00525-8)
Supplement: Supplementary file 4 — Additional file 4. Appendix 4: Patient scenarios presented with highlight elements aligning with triage tool. [file 13047_2022_525_MOESM4_ESM.pdf]

Appendix 4. Patient scenarios presented with highlight elements aligning with triage tool.

**Adult presentations**	Score on risk table	n (%) of 27 participants recommending face to face care provision
Bill is 54 and regular patient, attending every 12 weeks for skin and nail care. He lives with his wife. No medical history, gets a corn on his 5th toe when playing golf. He is due for 12 week appointment this week	0	3 (11%)
Jenny is a 23 police officer. She is new to the practice and referred from her GP. Referral states that she started her police role six weeks ago and has heel pain resulting in her missing one shift.	5	16 (69%)
Barbara, a 74 year old female returning patient, reports she is in pain with two new corns on the tops of her little toes following the purchase of new shoes. She has had these 12 months ago. The corns only hurt when she is wearing her new purchased sneakers but no other footwear. Medical history includes hypertension well managed by medication, diabetes managed with oral hypoglycemics, last HbA1c recorded as 7.2 mmol and a TPI 90 mmHg both feet.	4	10 (37%)
Tom, a 24 year old male who previously attended >12 months ago. Reports very sore toenail on his right big toe. Has a history of infected ingrown toenails due to often tearing his toenails. Medical history includes autism, he is able to communicate, but requires a carer to get to appointments and lives in an assisted living arrangement.	7	27 (100%)
Ngan is 18 and rings for an appointment as her toe got a little sore after a walk. She last attended 6 months ago with an infected ingrown toenail that cleared quickly after showing her how to self care. Medical history/meds: Oral contraceptive only.	1	7 (26%)
Chi is 56 and was discharged from hospital. He has a discharge summary from the High Risk Foot Clinic about monitoring his recently healed plantar foot ulcer on his right foot. Medical history/medications: Metformin, Insulin, catapres, his TPI are 0.5 Left/ 0.6 Right mmHg	8	26 (96%)
Dylan is 30 has a GP referral stating "foot pain" and no other medical concerns. Dylan is working from home and can sit most of the day. He reports the pain isn't interfering with work. Medical imaging indicated no known pathology.	0	1 (4%)
**Paediatric presentations**	Score on risk table	N (%) of 17 participants providing face to face care
Christos is 11. You saw him 12 months ago for calcaneal apophysitis which resolved with heel lifts and reduced activity. He has heel pain again in the same place, sometimes. He has no other medical history or medications.	0	1 (6%)
Jacinta is 3 and a NDIS participant. She walks well with her AFO's but recently started getting redness at her left ankle. This has blistered once	6	15 (88%)
A mum phones you regarding Bella who is unknown to the clinic. She is complaining about her right leg being sore. Parent reports she had a large splinter in her heel a few weeks, removed by GP under LA. Bella is now limping on the same leg.	9	16 (94%)
Marcus, is 6 year old child, referred by their specialist for urgent nail care 'as foster parents aren't comfortable doing it. Marcus has Level 3 autism spectrum disorder, uses a k-walker to ambulate and has a history of recurrent tinea infections.	5	15 (88%)
Farrah is 18 months. At the last face to face visit (2 months ago) the maternal and child health nurse suggested a podiatry assessment for intoe walking. There are no other concerns	1	3 (17%)
Ju is 18 months old. You saw her at 11 months for her parent concerns about her not walking. You monitored and she took independent steps at 13 months. Her parents have rung to tell you she is walking less recently in the afternoons.	4	6 (35%)
Jayden is 4 years old. His parents have rung for an appointment as they noticed when he is walking on the floor boards at home he is really loud and it looks like he has flat feet. His father has some foot problems and wears orthoses.	0	2 (12%)
